# The Influence of Minimum Sitting Period of the ActivPAL™ on the Measurement of Breaks in Sitting in Young Children

**DOI:** 10.1371/journal.pone.0071854

**Published:** 2013-08-14

**Authors:** Zubaida Alghaeed, John J. Reilly, Sebastien F. M. Chastin, Anne Martin, Gwyneth Davies, James Y. Paton

**Affiliations:** 1 School of Medicine, University of Glasgow, Glasgow, United Kingdom; 2 Physical Activity for Health Group, University of Strathclyde, Glasgow, United Kingdom; 3 School of Health and Social Care, Glasgow Caledonian University, Glasgow, United Kingdom; 4 Institute of Sport, PE and Health Science, University of Edinburgh, Edinburgh, United Kingdom; 5 National Heart and Lung Institute, Imperial College London, United Kingdom; University of Sao Paulo, Brazil

## Abstract

**Background:**

Sitting time and breaks in sitting influence cardio-metabolic health. New monitors (e.g. activPAL™) may be more accurate for measurement of sitting time and breaks in sitting although how to optimize measurement accuracy is not yet clear. One important issue is the minimum sitting/upright period (MSUP) to define a new posture. Using the activPAL™, we investigated the effect of variations in MSUP on total sitting time and breaks in sitting, and also determined the criterion validity of different activPAL™ settings for both constructs.

**Methods:**

We varied setting of MSUP in 23 children (mean (SD) age 4.5 y (0.7)) who wore activPAL™ (24 hr/d) for 5–7 d. We first studied activPAL™ using the default setting of 10 s MSUP and then reduced this to 5 s, 2 s and 1 s. In a second study, in a convenience sample of 30 pre-school children (mean age 4.1 y (SD 0.5)) we validated the activPAL™ measures of sitting time and breaks in sitting at different MSUP settings against direct observation.

**Results:**

Comparing settings of 10, 5, 2 and 1 s, there were no significant differences in sitting time (6.2 hr (1.0), 6.3 hr (1.0), 6.4 hr (1.0) and 6.3 hr (1.6), respectively) between settings but there were significant increases in the apparent number of breaks - (8(3), 14(2), 21(4) and 28 (6)/h) at 10, 5, 2 and 1 s settings, respectively. In comparison with direct observation, a 2 s setting had the smallest error relative to direct observation (95% limits of agreement: -14 to +17 sitting bouts/hr, mean difference 1.83, p = 0.2).

**Conclusion:**

With activPAL™, breaks in sitting, but not total sitting time, are highly sensitive to the setting of MSUP, with 2 s optimal for young children. The MSUP to define a new posture will need to be empirically determined if accurate measurements of number of breaks in sitting are to be obtained.

## Introduction

With increasing recognition that adults and children spend most of their waking hours sitting, research on time spent sitting, and its impact on health has proliferated in recent years [1], [2]. Evidence is also emerging that sitting periods interrupted frequently by periods of standing or activity may have different relationships with health outcomes than longer periods of uninterrupted sitting [3], [4]. Animal studies provide supportive evidence that prolonged periods of uninterrupted sitting are related to increased risk of cardio-metabolic disease [1], [3]. A recent review [5] concluded that the development of accurate methods for measurement of sitting time and breaks in sitting time was a high priority in sedentary behavior research.

Evidence from adults suggests that measurement of breaks in sitting may be less accurate with traditional accelerometers, such as the Actigraph, than with accelerometers designed specifically to measure posture and posture transitions such as the activPAL™ [6]. The activPAL™ has been validated for measurement of both sitting and breaks in sitting in adults [7].

As yet there has been little research on the possible health consequences of variations between individuals in breaks in sitting time in children. This is largely because there has been a lack of practical, objective and validated methods suitable for measuring sitting time and breaks in sitting in free-living children [8]. Kwon et al [9] and Mitchell et al [10] both reported, in longitudinal studies of older children and adolescents, that Actigraph determined breaks in sitting decline with age, but there is little evidence on the accuracy of the Actigraph for measurement of breaks in sitting [10]. Concurrent validity of the activPAL™ (against the Actigraph) for group-level estimates of total time sitting has been established for pre-school [11] and older children [12]. Criterion validity (against direct observation) of activPAL™ measurements of time spent sitting was also shown to be high in our previous study of pre-school children [13]. However, evidence on criterion validity of the number of breaks in sitting is less clear and may depend on activPAL™ settings.

In adults, the available evidence points to more frequent changes in posture being generally beneficial for health [3], [4]. Similar evidence is not yet available for children. However, the activPAL™ is an event-based monitor that samples at 10 Hz and could therefore capture very frequent changes in posture. It is not clear that events (postural changes) occurring at a frequency of ≤1 HZ (i.e. ≤1 change per sec) are likely to have any physiological meaning. In order to screen out very short events, the activPAL™ software includes an algorithm that only counts events *longer* than a specified duration, set by default at 10 s minimum sitting/upright period (MSUP) (i.e. ≥10 s of sitting/lying or upright data is needed to register as a new sitting/lying, or upright, event). In effect, this software setting determines the minimum period to define a new posture such as sitting [13], [14]. In many published studies, this setting has been left at a default value of 10 s as per the manufacturer’s specifications [15].

In a previous study in pre-school children, we changed the minimum sitting/upright period (MSUP) (which can be varied within the activPAL software from 1 s to 100 s) to 1 s because posture transitions appeared to be much more rapid in young children than in adults [13], [14]. Using this 1 s setting, we found that the activPAL™ provided accurate relative rank-ordered assessments of breaks in sitting, but significantly overestimated the number of breaks in sitting when compared to direct observation [14].

It is easy to imagine that the time required to transition from one posture to another e.g. sitting to standing might be different at different ages. A young child would be expected to change posture very quickly but an elderly person might take much longer. However, at present, the optimum activPAL™ setting of a MSUP to define a change in posture for measurement of sitting time and breaks in sitting is not known, either for early childhood or later in childhood or adult life. Furthermore, the effect of changes in the minimum period of sitting/upright on measurement accuracy of both sitting time and breaks in sitting time is unknown and has not been explicitly investigated. The present research, therefore, aimed to examine the effect of variations in the activPAL™ minimum time setting on both the total time spent sitting, and breaks in sitting (study 1), and to determine the criterion validity of different minimum event duration settings (study 2) using direct observation as the criterion method.

## Methods

### Ethics Statement

The University of Glasgow Medical Faculty Ethics Committee approved the study. Parents gave written informed consented to participation and children assented to the individual study procedures.

We used two groups of children to investigate the effects of changing minimum sitting/upright period. For both studies age and sex were recorded, height and weight were measured and body mass index (BMI) calculated. They were converted to z scores using the appropriate 1990 British growth reference [16], [17].

### Study 1

For the first study, the data were collected from a convenience sample of 23 healthy, free-living preschool children in Glasgow, Scotland. Information letters were distributed to head teachers of nurseries (N = 4) and local contacts (mainly colleagues with pre-school aged children). Parents who agreed to take part made an appointment with the researcher where written consent was obtained and baseline data and measurements completed. Each child was asked to wear an activPAL™ monitor (PAL Technologies, Glasgow, UK) continuously, 24 hrs a day, for between 5 to 7 days. The monitor was placed directly on to the skin of the child’s mid-thigh area using a small hypo-allergenic adhesive gel patch (PALstickies™), and was covered with a transparent sticky film (Tegaderm™) to secure it. As the device used was not waterproof, parents were asked to remove the monitor for any showering, bathing or swimming during the monitoring period. It was not routinely removed during the night. Parents were asked to note in a daily diary any time the device was removed as well as the time the device was reattached. For each child, periods noted in the daily diaries when the child was not wearing the device e.g. because of swimming, bathing/showering or delayed reattachment because of forgetting were identified and excluded from the raw activPAL™ files before analysis. During the period of monitoring, the children were attending nursery during weekdays and were taking part in normal nursery activities – in the classroom, during physical activity in nursery school and during periods of free play. All parents received verbal and written information and instructions about using the device before giving informed consent to the study.

For all children, the minimum duration of device wear time has been previously established as three weekdays with at least 6 hours of monitoring during waking hours per day [13]. In practice, in this study device wear time was much greater. In our final analysis, only weekdays were considered to avoid any effects arising out of different patterns of activity during weekend days.

### Data Reduction, Operationalization of Sitting Variables

The activPAL™ output classifies an individual’s activity into three categories: “sitting/lying”; “standing” (standing with no movement); and “walking” (movement from one place to another). In addition, the activPAL™ identifies and counts posture transitions (sit-to-stand and stand-to-sit).

In this study, sitting (sit/lie) was characterized in the following ways:

#### 1. Total time sitting

Waking time was defined from the first sit to stand transition in the morning, marking the fact that the child had woken. The researcher identified this transition by manual inspection of the event file produced using custom software (HSC PAL analysis software v 2.14) developed by by Dall and Granat at Glasgow Caledonian University. This software allows detailed analysis of the activPAL™ output as classified by the original activPAL™ Professional Research Edition software. The software generates a file listing the time (in seconds) at which a change in output category (i.e. a transition) occurred [13]. Arbitrarily, the end of waking time was standardized at 9 pm for all participants on all days of measurement. Total time recorded as “sit/lie” during waking hours was calculated. We also calculated the percentage of daily time spent in sit/lie and stand as recorded by activPAL™ during waking, as previous studies have included this as a measure of volume of sitting behavior [11].

#### 2. Breaks in sitting

The number and frequency of interruptions (“breaks”), defined as the number of transitions recorded from “sit/lie” posture to “stand” [18] during waking time were counted using the activity profile (summarized by hour) by activPAL™ Professional Research Edition software (Version 5.8.2.3). Only transitions from sit/lie to stand were counted and not stand to sit/lie transitions.

#### 3. Sitting bouts

We calculated the number and duration of each individual sitting bout, defined as duration in seconds spent “sit/lie” ending in a postural transition [19]. The number and duration of sitting bouts (sit/lie) were quantified using HSCPAL analysis software (version 2.14). [13], [14].

The length of individual sitting bouts and their distribution was represented by accumulation curves [19] and a fragmentation index [20]. Accumulation curves (Lorenz curves) characterize how an individual aggregates their sitting time [19], [21] and relate the amount of time accumulated in bouts shorter or equal to a given length. These curves can be reduced to single metrics at any point along the curve but the 50% and 90% points have been suggested to be the most interesting [21]. The fragmentation index (calculated as the number of sitting bouts/total sitting time measured in hours) [20] is a metric that summarizes information about breaks and the accumulation curves in one single metric. The fragmentation index (with units of number of bouts/total sitting hr) normalizes the number of breaks in sitting by removing the influence of total sitting time and provides a simple single measure of whether an individual accumulates their sitting time in a many short bouts or in a smaller number of longer bouts [20]. A higher fragmentation index indicates that time spent sitting is more fragmented with shorter sitting bouts. Both these approaches have been used to characterize sitting behavior in adults [19], [20].

As noted above, although the activPAL™ is an event based system, the analysis software only counts breaks in sitting lasting longer than a user defined MSUP in the new posture. This is intended to exclude very short postural “events” that are recorded by the monitor but are likely to have no physiological meaning, and is set by default at 10 s. In the present study, we systematically investigated the effect of reducing the MSUP from 10 s, through 5 s, 2 s, to 1 s within the activPAL™ software. Changing the MSUP in the activPAL™ software involves manually changing the setting in the range from 1 s to 100 s and only affects the time the monitor waits to decide whether a posture is seated or upright posture. Changing the MSUP has no effect on stepping time. Steps are detected directly using a different algorithm that does not take MSUP into account.

### Study 2

The second study was an assessment of the criterion validity of measurement of breaks in sitting in a different group of free-living pre-school children. They were a convenience sample of 32 pre-school children (4.1 y (0.5)) recruited from nursery schools in Scotland who were videoed for an hour while playing freely at nursery while wearing an ActivPAL™ monitor. Data analysis was performed on children (n = 30) with a complete data set for activPAL™ and direct observation outcomes. The study is described in detail elsewhere [13], [14] but in brief, each child wore an activPAL™ monitor and simultaneously was filmed for 1 hour during their usual activity in nursery. Second by second direct analysis of the video was then used to count number of breaks in sitting time.

In study 2, the raw activPAL™ files were reprocessed using MSUP of 2 s, 5 s and 10 s and for each child, the number and duration of sit/lie periods was calculated from direct observation files and was compared with the activPAL™ analyses using the varying settings.

Both studies used activPAL™ Professional Research Edition software (Version 5.8.2.3).

In both studies, the activPAL™ HSCPAL software files and the activPAL™ pal files (activity profile summarized by hour) were used in our data analysis. We made no use of the “15 s epoch file” in the available activPAL™ software.

### Statistical Analysis and Study Power

Statistical analyses and calculations were conducted using the Minitab statistical software version 16.1 (State College, PA, USA) and Microsoft® Office Excel 2007. For both studies, a convenience sample of around 20 children was deemed a priori, likely to be sufficient to characterize differences in the number of posture transitions, as measured between 1 s and 10 s minimum time spent sitting settings. Preliminary analysis of 20 sets of paired activPAL™ data (i.e. 10 s and 1 s data from the same child) in study 1 showed that the difference in number of posture transitions measured by the 10 s and 1 s setting was highly statistically significant and so only those children recruited to the study at that point were included and no further recruitment took place. Paired t tests were used to test the significance of differences in variables measured. Repeated measures Analysis of variance (ANOVA) using Tukey’s correction for multiple comparison was applied to compare the mean values for each MSUP. A Bland-Altman analysis for assessing agreement between two measurements [22] was carried out. The limits of agreement between the number of sitting bouts during direct observation (criterion method) vs sitting bouts calculated by the activPAL™ using different MSUP activPAL™ settings (1 s, 2 s, 5 s and 10 s minimum sitting/upright period) were set at mean difference +/−1.96 x standard deviation (SD). (The graph for the 1 s comparison has previously been previously published [13]). The pattern of accumulation of sitting bouts by direct observation data and activPAL™ data with different settings (1 s, 2 s, 5 s &10 s) was represented by accumulation curves [19]. All variables were checked for normal distribution and means and SDs were used to summarize normally distributed values. For all tests, significance was taken at p = 0.05.

## Results

### Characteristics of Study Participants

#### Study 1

Of the 23 children recruited to study 1, 20 provided adequate data of at least 3 days (3 children wore the monitors less than 3days during the study) [11], 9 boys and 11 girls; mean age 4.5 (SD 0.7); mean height 107.7 cm (4.9), mean weight 19.6 kg (3.9) and mean body mass index 16.6 kg/m^2^ (2.0). The mean z-scores were 0.24 for height, 0.60 for weight and the median z-score 0.16 for body mass index (BMI). Mean (SD) monitoring time was 3.8 d (0.7), 22.3 hr (1.5) per 24 hr period, of which a mean of 11.9 hr (1.0) was in waking hours. Missing data (where monitor was removed because of swimming, bathing/showering or monitor and not reattached according to parent’s record) accounted for a mean of 5.1% (SD 3.4) of total monitoring hours.

### Breaks in Sitting in Free-living Children

#### Study 1

**Figure 1:**
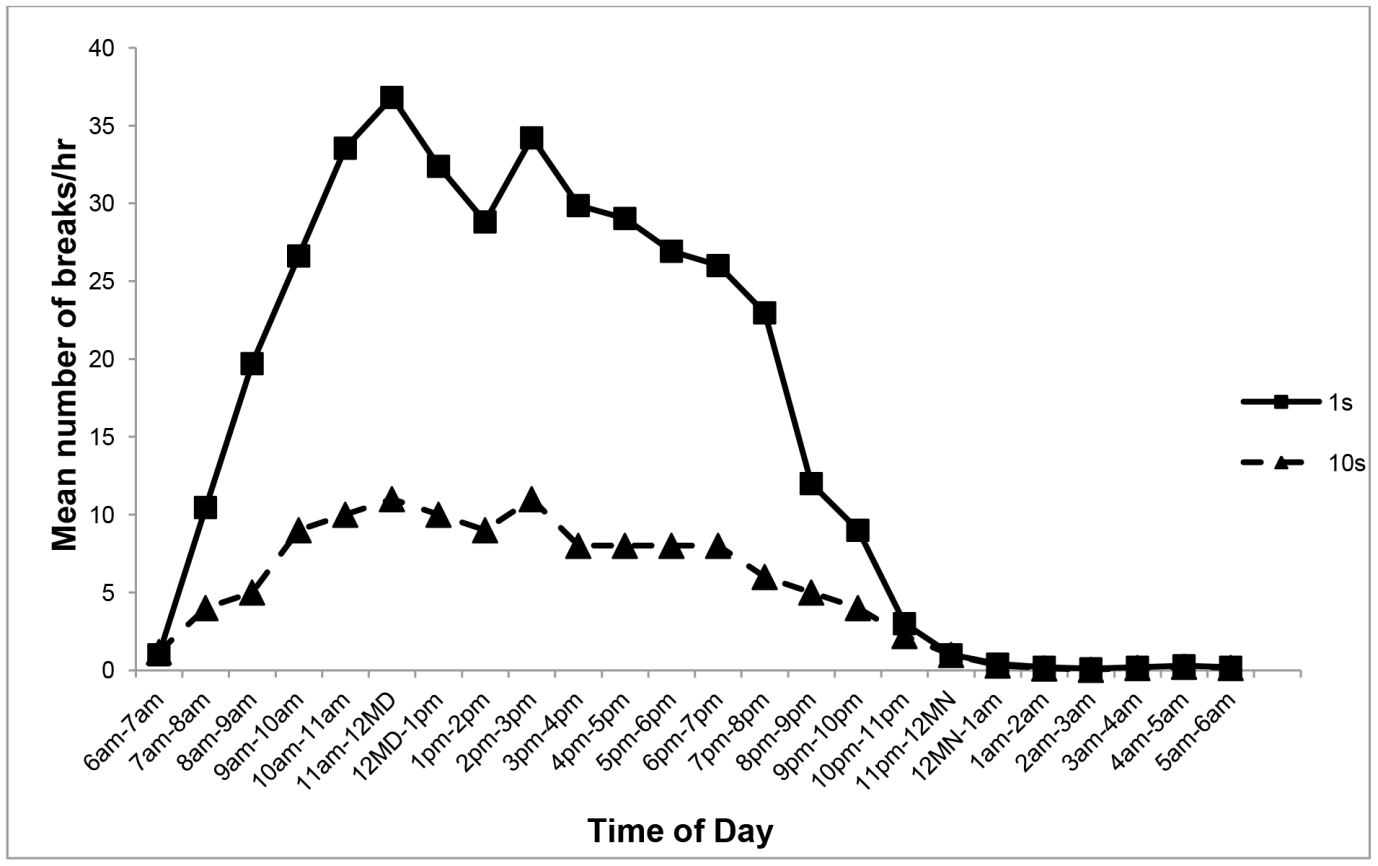
The mean number of breaks in sitting/hr during 24 hr using minimum time to define new position settings of 10 s and 1 s. Study 1(n = 20).

A plot of number of breaks in sitting per hour against time during 24 hours is shown in figure 1 for both day and night hours using 10 s vs 1 s MSUP. There was a gradual increase in the number of breaks per hour from morning until afternoon with a dip after lunch-time and a peak at around 4 pm followed by a decrease in the evening until the child went to sleep (Figure 1). During the night, generally no breaks were recorded from midnight until early morning. However, occasionally a few breaks occurred between 9 pm and 12midnight (Figure 1).

In the light of the above, the rest of the analysis was restricted to breaks in sitting during waking hours. Using a minimum activPAL™ sitting/upright period of 10 s, the mean (SD) percentage of waking time spent sitting was 52.3 (6.2) %. The total sitting time was 6.2 hr, (1.0) during waking hours (11.9 hr (1.0)). The total number of breaks in sitting during waking hours was 109 (18) giving a mean number of breaks of 8 (3) per hour. Using a 10 s MSUP, around 90% of sitting bouts during waking hours were ≤8 min (1.5) and the mean (SD) fragmentation index (number of bouts/total sitting time (hr)) [20] during waking hours was 19.3 (3.7) (Table (1).

**Table 1:** Description of sitting behaviors *during waking hours* +, mean (SD) for study 1 (n = 20).

	10 s setting (Default)Mean (SD)	5 s settingMean (SD)	2 s setting Mean (SD)		1 s settingMean (SD)	p-value
**Total sitting time (hr)**	6.2(1.0)	6.3(1.0)	6.4(1.0)		6.3(1.6)	0.9
**% Sitting time (defined as-sit/lie only)**	52.3 (6.2)	52.5 (5.9)	53.5 (5.4)		52.9 (6.3)	0.7
**% Sitting time (defined as sit/lie and** **quiet standing)**	80.1(8.3)	80.3 (4.6)		82.1 (3.9)	81.5(8.9)	0.5
**Number of breaks (sit to stand)/hr** *	8 (3)	14 (2)		21(4)	28 (6)	0.00
**Total number of breaks in** **sitting (transitions)** *	109 (18)	173 (43)		278 (78)	376(90)	0.00
**Number of sitting bouts** &	118 (18)	182 (28)		289 (52)	382 (80)	0.00
**50% sitting bout length**	80 s (14.7)	55 s (4.2)		50 s (4.2)	42 s (7.7)	0.00
**90% sitting bout length**	8 min (1.5)	6 min (1.1)	3 min (1.0)		60 s (10.4)	0.00
**Fragmentation index**	19.3 (3.7)	29 (5.0)	46 (9.0)		61.6 (16.4)	0.00

+ Waking hours were defined as “From the first sit to stand transition in the morning to 9 pm”.

* Calculated from activity profile summarized by hour using activPAL™ Professional Research Edition (Version 5.8.2.3).

& Calculated using activPAL™ HSCPAL analysis software (version 2.14). The PAL files generated by the activPAL™ software were imported into HSC PAL analysis software (developed by Dall and Granat).

### The Difference in Estimated Sitting Time and Breaks in Sitting using 10 s vs 1 s MSUP

#### Study 1

The measures of sitting time during waking hours with the different MSUPs are shown in Table 1. There were no significant differences in the mean sitting time when expressed either as total time measured in hours or as a percentage (6.2 hrs (52.3%) vs 6.3 (52.9%), Paired t test p = 0.45) (Table 1).

However, for the number of breaks, number of bouts, bout periods and fragmentation index (number of bouts/sitting hour) there were significant differences as the MSUP was varied. Changing from a 10 s setting to a 1 s setting for MSUP led to significant increases in: the total number of breaks in sitting (109 (18) vs. 376 (90), p = 0.001); the number of breaks per hour (8 (3) vs. 28 (6), p = 0.0001); and the total number of sitting bouts (118 (18) vs. 382 (80), p* = *0.0001). Around 90% of sitting bouts were ≤8 min using a 10 s setting but were ≤1 min using a 1 s setting. The fragmentation index using a 1 s setting was nearly 3 times greater than when using 10 s setting: (61.6 (16.4) vs. 19.3 (3.7), p = 0.001) consistent with more fragmented, and shorter sitting bouts.

In addition, marked inter-individual differences in the pattern of accumulation of sitting bouts were observed. As an example, in 2 children, where the total time sitting of both children was similar at 53% and 55% of 10.2 h (0.9) and 10.7 h (1) waking hours respectively, the accumulation of their bouts was different: using a 10 s minimum sitting/upright period, 90% of sitting bouts were ≤6 min in child 1 and ≤15 min in child 2 and about 50% sitting bouts were ≤55 s in child 1 and ≤75 s in child 2. Moreover, child 1 had a fragmentation index of 17.9 (5.2) and 82.5 (20) with 10 s and 1 s settings respectively, and child 2 had a fragmentation index of 15.8 (4.2) and 37.3 (10.2) with 10 s and 1 s respectively.

#### Study 2

Thirty preschool children completed simultaneous activPAL™ and direct observation monitoring (10 boys and 20 girls; mean age 4.1 y (0.5), mean height 105.1 cm (5.1), mean weight 18.7 kg (3.8) with a mean body mass index 16.8 kg/m2 (2.1). The mean z-scores were 0.64 for height, 0.79 for weight and 0.60 for BMI. A total of 16167 s (14.2%) was ‘off screen’ time from 113,917 total measured seconds for the 30 children [13], [14].

In study 2, combining data from all participants (n = 30), the total time spent sitting during direct observation were compared with the total time spent sitting using the activPAL™ setting 2 s, 5 s and 10 s MSUP. The total time spent sitting was 12.5 hr during direct observation and 11.3 hr with 1 s MSUP [14]. With 2 s, 5 s and 10 s the total sitting time was 11.4 hr, 11.2 hr and 11.3 hr, respectively.

**Figure 2:**
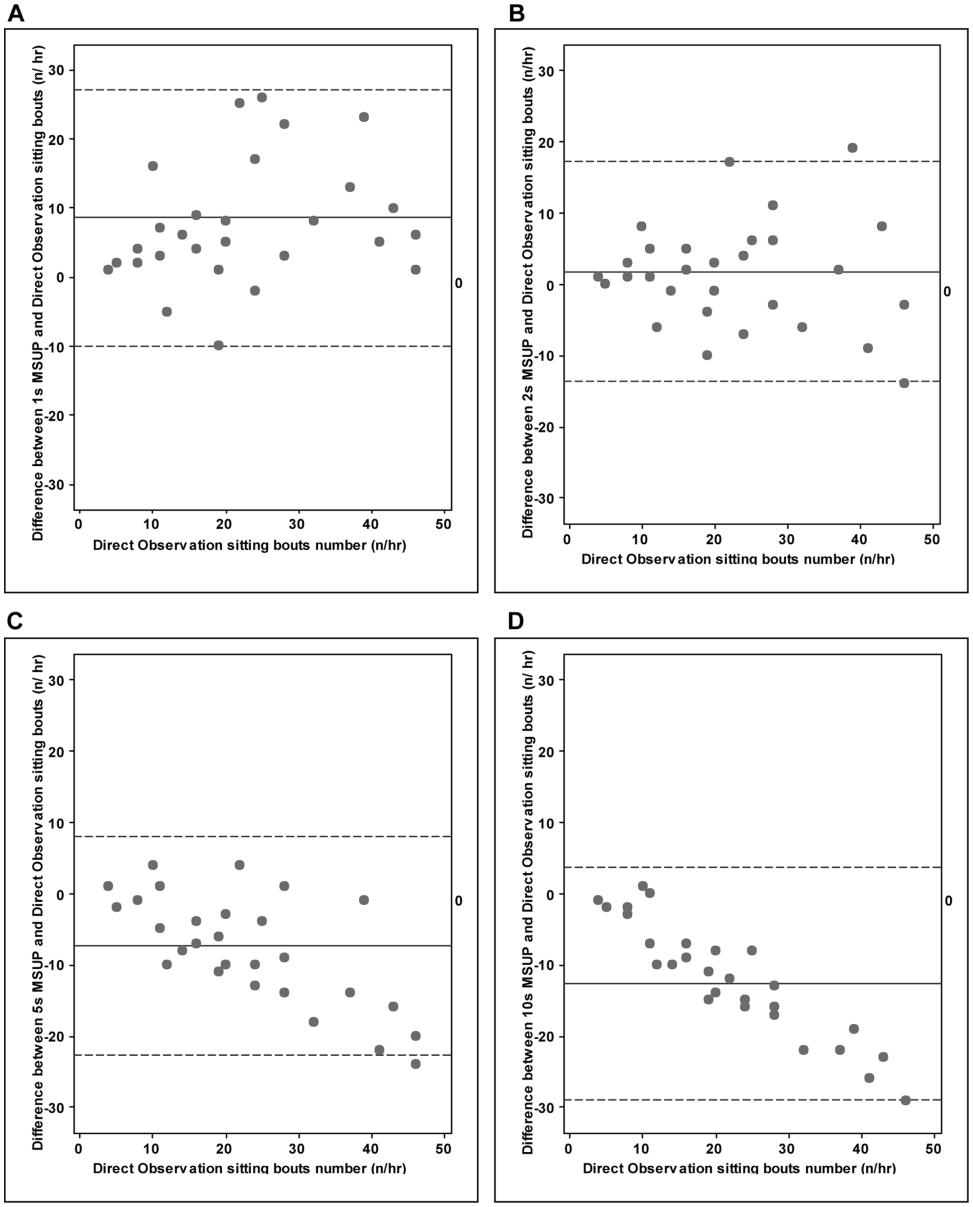
Individual Bland-Altman plots comparing the difference in number of sitting bouts during direct video observation (direct observation) with the number of sittings bouts measured by the activPAL™ with different activPAL™ settings for minimum sitting/upright period (1s – diff1 (A), 2s – diff2 (B), 5s - diff5 (C) and 10s - diff10s (D)). Study 2 (n = 30). Data for 1 s taken from Davies et al [15]. Direct Observation is considered the criterion or gold-standard and it is used on the x-axis. Mean bias is represented by a solid line, 95% limits of agreement by dashed lines.

For bouts of sitting, the average number of bouts per hr using direct observation was compared with bouts measured simultaneously using the activPAL™ using 2 s, 5 s and 10 s MSUP respectively. Figure 2 shows Bland-Altman plots comparing the different numbers of sitting bouts during direct observation vs different MSUP of 1 s, 2 s, 5 s and 10 s on the activPAL™ for each child (n = 30) are shown. It can be seen that the use of a 2 s setting for activPAL™ MSUP minimized bias and showed no significant difference relative to direct observation (limits of agreement -14 to +17 bouts per hr, mean difference 1.83, paired t-test p = 0.2). However, the 5 s and 10 s settings underestimated the number of sitting bouts as measured by direct observation (for 5 s limits of agreement -23 to 8, mean difference -7.27 and for 10 s limits of agreement -29 to 4, mean difference -12.57, paired t-test p = 0.001, respectively). While the bias is much smaller with a 2 s setting the limits of agreement are quite wide, and of similar magnitude to the other settings. This means that the average with a 2 s setting will be more accurate, but for any individual the errors with 2 s will be nearly as large as for the other settings.

**Figure 3:**
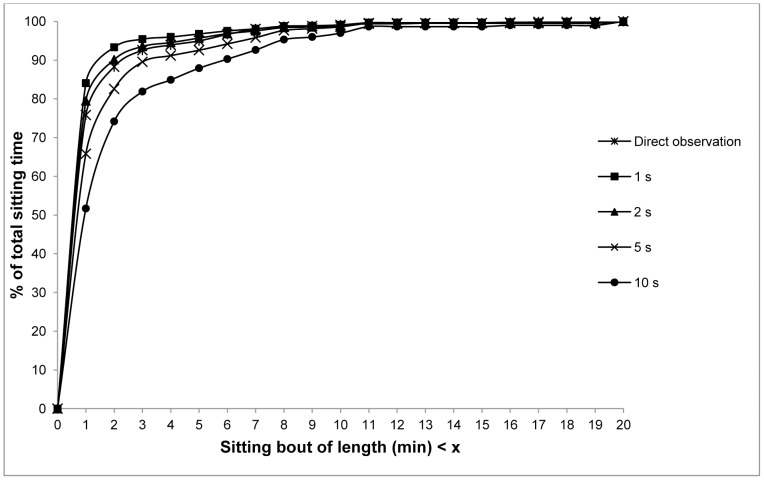
The pattern of accumulation of sitting bouts during direct observation, using minimum sitting/upright periods of 1s, 2s, 5s and 10s. Study 2 (n = 30). The numbers on the x-axis are cumulative – hence the y intercept at a particular x value represents the number of sitting bouts occurring shorter than or equal to a given x axis value.

Figure 3 shows the pattern of accumulation of sitting bouts during direct observation with 1 s, 2 s, 5 s and 10 s MSUP. 90% of sitting bouts were identical (at ≤2 min) for both direct observation and from the activPAL™ using the 2 s MSUP.

## Discussion

### Main Findings and Study Implications

This is the first study to examine the effect of varying the activPAL™ MSUP to define a new posture setting on measurements of total time spent sitting and breaks in sitting. In study 1 we showed that varying the activPAL™ setting had only a negligible impact on measurement of total time spent sitting. However and in contrast, for breaks in sitting there is a marked difference varying systematically with the setting used.

In study 2, we showed that the systematic differences in measures of breaks in sitting described in study 1 have an impact on the accuracy of the measurement of breaks in sitting. The result is that a default setting of 10 s for the activPAL™ appears unsuitable for quantification of breaks in sitting in young children, in whom a minimum sitting/upright period of 2 s will provide much higher accuracy with minimal bias.

The present study also shows that important features of sitting behavior in young children can be characterized objectively by a few fundamental metrics: volume of sitting; frequency of breaks in sitting; and pattern of accumulation of sitting bouts as represented by accumulation curves and a fragmentation index [21]. Using a 2 s MSUP, the mean volume of sitting in study 1 was 6.4 hr (1.0) during waking hours, the number of breaks in sitting around 21/hr (4), the fragmentation index 46(9.0), and 50% of sitting bouts were less than 50 s (4.2) (Table1).

Healy et al [4] have previously reported in adults that increased breaks in sitting time (resulting in short sitting bouts) are associated with better metabolic health, a relationship that was independent of total sitting time. The fact that sitting behaviors can be characterized objectively by a few simple measures means that comparative studies investigating the longer-term health effects can now be undertaken in children.

### Comparisons with Other Studies

To our knowledge, no previous studies have examined the effect of the activPAL™ MSUP to define a new posture on measurements of sitting time and breaks in sitting.

In general, other studies have not commented on what setting was used to define minimum sitting/upright period. As an example, Lyden et al in a recent activPAL™ validation study (against direct observation) of 13 free-living adults monitored for about 10 consecutive hours on 2 separate days [4 M, 9 F; mean age 24.8 (5.2)] reported that the activPAL™ was a suitable tool to measure breaks in sitting in this older age group with 5.1 (range 2.8–7.1) breaks in sitting per sitting hour [6]. This study did not specify whether the default 10 s MSUP was used.

Harrington et al noted the mean length of sitting bouts in adolescent females using activPAL™ was 9.8 (0.2) minutes [18]. Harrington used a customized MATLAB programme to process the activPAL™ data output files. This examined each epoch which contained a full 15 s of sitting/lying and classified this as the beginning of a sitting bout which continued until the next 15 s bout of standing or stepping was identified. Chastin and Granat using the activPAL™ with a 10 s MSUP found that the mean sitting bout length in free-living adults was 45 minutes [19]. In contrast, and using a 10 s minimum sitting time for purposes of comparison, the majority of sitting bouts for the young children in the present study (study 1) lasted ≤8 minutes suggesting that the children studied predominantly accumulated their sitting time in short bouts.

Studies using objective measures of fragmentation index are non-existent in children and scarce in adults. A recent study in 30 healthy adults (using activPAL™ continuously over 7days) found that the mean fragmentation index [bouts/sitting time (including sleeping time) (hr)] in men was 2.6(0.8) and 3.3(0.4) in women [20]. In the present study, the mean (SD) fragmentation index (again using the default 10 s MSUP for comparison) was much higher, 19.3(3.7). Our present study is not directly comparable because we excluded sleeping time, where subjects would be expected to have no postural transitions. A preliminary reanalysis of 3 subjects in the present study, chosen at random, showed that even including sleeping time the fragmentation index is about 3 times greater than that reported by Chastin et al. Our evidence, therefore, suggests that young children have much more fragmented sitting time with a pattern of shorter sitting bouts interrupted by more frequently by breaks.

Because of its impact on the measurement of breaks in sitting and other measures such as fragmentation index, the present study suggests that more attention must be paid to this instrument setting. It seems intuitively likely that the most suitable setting for measurement of breaks in sitting time may vary with age. We would hypothesize that children can transition to a new posture more frequently than adults, and the optimum setting for measurement in breaks in sitting may lengthen as subjects get older. We suspect that it is likely that empirical studies using the activPAL™, or other similar event based monitoring systems, will in future be required to define the best setting for minimum duration of sitting for each age.

### Study Strengths and Limitations

The present study does not assess the biological importance of sitting time or fragmentation, but that was not the aim of the present study. Methodological evidence aimed at the establishment of accurate yet simple and objective measures for characterizing sitting time and fragmentation will be fundamental to future studies which try to relate these constructs to health outcomes, and essential for evaluation of future intervention studies.

Previous studies of movement in young children and adults, particularly those using the Actigraph monitor, have used an analytical approach based on the analysis of sitting in 15 s epochs [12], [18]. A detailed comparison of the impact of different MSUPs in an event based analysis, as in our study, vs a 15 s epoch approach is beyond the scope of this paper. However, a preliminary analysis of 10 files from study 2 using the 15 s epoch file analysis present in the activPAL™ software showed that there were few changes when we used different MSUP settings and these were statistically not significant for either total sitting time or number of sedentary bouts.

### Conclusion

This study has established that the setting of MSUP to define a new posture has a significant impact on measurement of breaks in sitting in young children but not the measurement of total sitting time. In the age group we studied, 2 s appears to be an appropriate minimum sitting/upright period to define breaks in sitting using the activPAL™. It is probable that the optimum instrument setting for minimum sitting/upright period will be different at different ages. Standardization of the technical aspects of measurement and of measures to describe sitting time will allow longer term studies of the health effects of sitting behaviors as well as providing comparable baseline data for intervention studies.
